# Comparative Trial of Continuous Flow Enteral and Intravenous Fluid Therapy in Horses

**DOI:** 10.3389/fvets.2021.686425

**Published:** 2021-08-06

**Authors:** Domingos C. R. Dias, José D. Ribeiro Filho, Rinaldo B. Viana, Thereza C. C. Bittencourt, Fernanda T. D. R. Dantas, Raffaella B. C. Teixeira, Paula A. Di Filippo, Hélio C. Manso Filho, Samuel R. Alves, Paulo V. M. Santos, Nadyne S. Moreira

**Affiliations:** ^1^College of Veterinary Medicine and Animal Science, Universidade Federal da Bahia, Salvador, Brazil; ^2^Laboratory of Research in Veterinary Internal Medicine, Veterinary Department, Universidade Federal de Viçosa, Viçosa, Brazil; ^3^Institute of Animal Health and Production, Universidade Federal Rural da Amazônia, Belém, Brazil; ^4^Crescer Consultancy Service, Salvador, Brazil; ^5^Laboratory of Animal Clinic and Surgery Center of Agricultural and Livestock Sciences and Technology, Universidade Estadual do Norte Fluminense, Campos dos Goytacazes, Brazil; ^6^Zootechnics Department, Equine Researcher Center, Universidade Federal Rural de Pernambuco, Recife, Brazil

**Keywords:** equine, dehydration, rehydration, enteral fluid therapy, intravenous fluid therapy, isotonic, hypotonic

## Abstract

Continuous flow enteral fluid therapy with isotonic and hypotonic enteral electrolyte solutions are as safe and effective as intravenous fluid therapy. The aim of this study was to carry out a comparative assessment between continuous flow enteral and intravenous (IV) fluid therapy in adult experimentally dehydrated horses. Six experimentally dehydrated adult mares were used in a study carried out in a 6 × 3 crossover design, which each animal received three different treatments (isotonic enteral fluid therapy—EsISO, hypotonic enteral fluid therapy—EsHYPO and intravenous fluid therapy with Lactate Ringer Solution—LR IV, all in continuous flow). Solutions were administered at a rate of 15 mL^−1^.kg^−1^.h^−1^ for 8 h, after 36 h of water and food deprivation. Serum and urinary biochemical assessment; urinary volume, pH and specific gravity; and blood gas analysis were measured at −36, 0, 2, 4, 6, and 8 h. The dehydration period (DP) caused discrete hydroelectrolytic and acid base imbalances. The EsISO, EsHYPO and LR IV increased blood volume. Enteral solutions restored the imbalances yielded by the DP and all treatments increased urine volume. Also, the EsHYPO and LR IV showed no effects in acid base balance, while EsISO showed slightly acidifying effect. The present study certifies the efficacy and safety of isotonic and hypotonic continuous flow enteral fluid therapy in comparison to IV fluid therapy in dehydrated horses.

## Introduction

The therapeutic choice to regulate water, electrolyte and acid-base imbalances in horses is fluid therapy. Solutions containing electrolytes and other elements (such as energy sources) are administered to restore blood volume and perfusion in addition to rehydrate tissues and digesta. Among the different modalities of hydroelectrolytic therapies, the intravenous (IV) administration of commercial electrolyte solutions is traditionally considered the most effective (1) and also the most employed in equine medicine.

The administration of electrolytic solutions through the nasogastric route is also routinely used in horses, but to a lesser extent when compared to the intravenous modality. Enteral fluid therapy (EFT) via the nasogastric route has been studied with excellent results in the equine species (2–4). In EFT electrolyte solutions are infused directly into the gastrointestinal tract in order to be absorbed towards the plasma compartment.

In cases that are not severely dehydrated or at risk of hypovolemic or endotoxic shock, EFT has been shown to be as efficient as IV fluid therapy. In addition to being easily applicable, it allows modifications in the composition of electrolyte solutions in order to satisfy the needs of each patient, improving the efficacy of the solutions and enhancing the success rate of this therapy (5).

In recent years, EFT started to be administered by continuous flow (EFTcf). In this modality, electrolyte solutions are administered continuously for prolonged periods through small gauge nasogastric or nasoesophageal tubes. Thus, daily infusion of large volumes of solutions is possible without triggering any kind of discomfort—neither by the presence of the tube itself, nor due to abdominal distention. This has been proved true in the authors' clinical experience after years using EFTcf. Despite all that, this fluid therapy method is not widespread, remaining as a therapeutic modality restricted to Veterinary Hospitals.

To date, only one study (6) has been conducted comparing the use of intravenous Ringer's lactate solution with EFT. The aim of this study was to compare continuous flow enteral fluid therapy and IV fluid therapy in experimentally dehydrated adult horses. Our hypothesis was that EFTcf with isotonic and hypotonic electrolyte solutions is as effective as IV fluid therapy in rehydrating horses.

## Materials and Methods

Six non-pregnant Warmblood mares, with an average age of 10 ± 3 years and median body weight of 436 ± 40 kg, were used in the present study. All horses were considered healthy based on clinical and laboratory tests. Animals were housed in individual stalls, fed Tifton grass hay (Cynodon sp.), commercial concentrate (1 to 2% of body weight divided into 3 meals) and allowed free access to water and mineral supplement *ad libitum*. The experimental design was a 6 × 3 crossover. Each mare received all three treatments, one at a time in a rotational system with a washout period of 8 days. The mares were experimentally dehydrated prior to the fluid therapy phase. The dehydrating period (DP) consisted of 36 h of food and water deprivation associated with the administration of two doses of furosemide (1 mg kg^−1^, 12 h apart, IV). After the DP the animals received a hypotonic (EsHYPO) or an isotonic enteral electrolyte solution (EsISO) through a nasogastric tube (5 mm of internal diameter x 7 mm of external diameter and 1.5 m in length) or lactated Ringer's solution (LR IV) intravenously through the jugular vein with a 14-gauge catheter, all in continuous flow at a rate of 15 ml kg^−1^ h^−1^ for 8 h (fluid therapy period). The presence of the nasogastric tube in the stomach was confirmed by the return of gastric contents after aspiration.

The concentrations of each electrolyte solution used are described (Table 1).

**Table 1:** Composition of enteral and intravenous electrolyte solutions.

**Components**	**EsHYPO**	**EsISO**	**LR IV**
Sodium chloride	4 g	5 g	6 g
Potassium chloride	0.5 g	0.5 g	300 mg
Calcium acetate	1 g	1 g	---
Calcium chloride	---	---	200 mg
Magnesium chloride	0.2 g	0.2 g	---
Sodium lactate	---	---	3.1 g
Dextrose	---	10 g	---
Maltodextrin	5 g	---	---
Osmolarity	190 mOsm L^−1^	280 mOsm L^−1^	274.1 mOsm L^−1^

*EsHYPO, hypotonic enteral electrolyte solution; EsISO, isotonic enteral electrolyte solution; LR IV, intravenous lactated Ringer's solution (Fresenius Kabi Brasil Ltda. – Barueri – SP)*.

Blood samples were collected immediately before the start of the dehydration protocol (T-36 h, baseline values), at the end of the DP (immediately before the start of treatments) (T0h), two (T2h), four (T4h), six (T6h), and 8 h (T8h) after the beginning of treatments. Blood was collected for biochemical tests by jugular venipuncture using Vacutainer BD vials (Curitiba - PR - Brasil). To obtain serum, blood samples were collected in Vacutainer BD® tubes without anticoagulant and with clot activator and kept in a water bath at 37°C for 40 min, for clot formation. Serum separation was performed by centrifugation and then stored at −20°C until analysis. Chloride, magnesium, phosphorus, urea and creatinine concentrations were determined in serum samples by colorimetric enzymatic assay in the automatic biochemistry device Bioplus Bio 2000 (Laboratory Products Bioplus, Barueri-SP). Plasma glucose and lactate were determined on the same device. Serum osmolarity was determined by freezing point depression (Osmometer 3320, Advanced Instruments Inc, Massachusetts, USA).

Sodium, potassium, ionized calcium, and blood gas parameters (pH, pCO_2_, $\text{HC}{\text{O}}_{3}^{-}$ and Base Excess) were evaluated on i-STAT Handheld Blood Analyzer (Abbot Point of Care Inc, Princeton, USA), by collecting 2 ml of blood from the jugular vein with a BD® needle and heparinized syringe immediately after the blood sample collection. Anion Gap (AG) was calculated using the equation: AG = (Na^+^ + K^+^)–(Cl^−^ + $\text{HC}{\text{O}}_{3}^{-}$), while the strong ions difference (SID) was based on the formula SID = [(Na^+^ + K^+^)–(Cl^−^)] (7).

Urine volume was measured at every experimental time from T0h to T8h through a graduated collection bag connected to a 24-gauge Foley Rusch (3). Urine aliquots were collected to determine specific gravity through refractometry (Refractometer—Model 8494) and pH and glucose on a reagent strip (Combur Test—Roche). Urinary chloride, calcium, magnesium, urea and creatinine were measured by biochemical analysis (HumaStar 300 Automated Chemistry Analyzer, Human Diagnostics, Wiesbaden, Germany), while urinary sodium and potassium were determined by flame photometry (Photometer B462, Micronal, São Paulo, Brazil).

Results were analyzed using the statistical program SPSS 13.0. Quantitative variables were subjected to the Normality (Shapiro-wilk) and Homoscedasticity (Cochran) tests to verify if ANOVA requirements were met. If so, an analysis of variance based on planning of repeated measures was used to assess the effect of treatments, that is, each treatment at different times. The influence of time and the interaction between treatment and time were also analyzed. When the analysis showed a significant effect for one or more factors, Tukey test was applied to compare any and all contrasts between two treatment averages. All analyses were interpreted considering a significance level of 5%. When normality criteria were not met, differences between treatments in the same period were assessed using the Kruskal—Wallis test and differences within a treatment at different times using the Friedman test.

## Results

The DP did not affect serum sodium levels. During the fluid therapy phase, significantly higher sodium levels were observed in the LR IV treatment group (T2h, T4h, and T6h) in comparison to EsISO and EsHYPO (Table 2).

**Table 2:** Means and standard deviations of serum sodium (mmol L^−1^), potassium (mmol L^−1^), chloride (mmol L^−1^), ionized calcium (mmol L^−1^), magnesium (mg dl^−1^), and phosphorus (mg dl^−1^) in horses submitted to water and food restriction and treated in a 6 × 3 crossover design with different hydroelectrolytic therapy modalities.

**Treatment**	**Time**					
	**(-)36 h**	**0 h**	**2 h**	**4 h**	**6 h**	**8 h**
**Serum sodium**						
EsHYPO	134.00^Aa^ ± 2.53	133.17^Aa^ ± 5.53	130.17^Aa^ ± 5.34	130.00^Aa^ ± 4.47	131.33^Aa^ ± 4.37	134.00^Aa^ ± 4.56
EsISO	134.17^Aa^ ± 4.88	133.67^Aa^ ± 3.33	131.00^Aa^ ± 2.53	130.67^Aa^ ± 1.75	132.50^Aa^ ± 1.87	134.50^Aa^ ± 3.27
LR IV	133.17^Ba^ ± 5.56	134.17^Ba^ ± 4.83	134.67^Aa^ ± 4.72	134.83^Aa^ ± 4.58	135.17^Aa^ ± 4.22	134.67^Ba^ ± 4.50
**Serum potassium**						
EsHYPO	4.30^Aa^ ± 0.37	3.38^Ab^ ± 0.31	3.55^Ab^ ± 0.29	3.75^Ab^ ± 0.26	3.85^Aa^ ± 0.19	3.93^Aa^ ± 0.15
EsISO	4.13^Aa^ ± 0.85	3.50^Ab^ ± 0.32	3.53^Ab^ ± 0.28	3.57^Ab^ ± 0.22	3.73^Ba^ ± 0.23	3.85^Aa^ ± 0.15
LR IV	3.90^Aa^ ± 0.35	3.47^Ab^ ± 0.34	3.50^Ab^ ± 0.33	3.48^Ab^ ± 0.25	3.55^Ba^ ± 0.19	3.65^Aa^ ± 0.25
**Serum chloride**						
EsHYPO	98.50^Ab^ ± 4.32	94.83^Ac^ ± 3.66	96.83^Ac^ ± 3.19	97.17^Ab^ ± 3.13	104.00^Aa^ ± 3.58	101.17^Bb^ ± 4.31
EsISO	101.00^Ab^ ± 3.69	95.83^Ac^ ± 5.74	98.67^Ac^ ± 2.42	100.33^Ab^ ± 3.20	102.00^Aa^ ± 2.37	105.50^Aa^ ± 3.27
LR IV	99.50^Aa^ ± 2.43	94.33^Ab^ ± 1.75	97.50^Ab^ ± 3.08	98.33^Aa^ ± 1.75	98.17^Ba^ ± 2.56	100.83^Ba^ ± 2.56
**Serum ionized calcium**						
EsHYPO	1.75^Aa^ ± 0.07	1.56^Ab^ ± 0.16	1.84^Aa^ ± 0.08	1.86^Aa^ ± 0.18	1.81^Aa^ ± 0.12	1.78^Aa^ ±0.12
EsISO	1.67^Aa^ ± 0.08	1.51^Ab^ ± 0.13	1.80^Aa^ ± 0.14	1.85^Aa^ ± 0.14	1.81^Aa^ ± 0.13	1.80^Aa^ ± 0.11
LR IV	1.73^Aa^ ± 0.08	1.47^Ab^ ± 0.15	1.47^Bb^ ± 0.13	1.50^Bb^ ± 0.15	1.50^Bb^ ± 0.14	1.52^Bb^ ± 0.16
**Serum magnesium**						
EsHYPO	1.68^Ab^ ± 0.19	2.13^Aa^ ± 0.23	1.65^Bb^ ± 0.32	1.47^Ac^ ± 0.19	1.38^Ac^ ± 0.12	1.28^Ad^ ± 0.08
EsISO	1.85^Ab^ ± 0.36	2.25^Aa^ ± 0.22	1.95^Ab^ ± 0.28	1.58^Ac^ ± 0.29	1.45^Ac^ ± 0.24	1.40^Ac^ ± 0.43
LR IV	1.65^Ab^ ± 0.14	1.98^Aa^ ± 0.15	1.20^Cc^ ± 0.14	1.03^Bd^ ± 0.14	1.03^Bd^ ± 0.20	0.85^Be^ ± 0.10
**Serum phosphorus**						
EsHYPO	1.87^Ab^ ± 0.74	2.88^Aa^ ± 0.84	2.57^Aa^ ± 1.03	2.37^Aa^ ± 0.92	2.27^Ba^ ± 0.73	2.05^Bb^ ± 0.41
EsISO	1.77^Ab^ ± 0.60	2.98^Aa^ ± 0.73	1.88^Ab^ ± 0.69	1.65^Ab^ ± 0.52	1.57^Cb^ ± 0.58	1.47^Cb^ ± 0.56
LR IV	1.92^Ab^ ± 0.57	3.32^Aa^ ± 0.51	2.90^Aa^ ± 1.09	2.50^Ac^ ± 0.69	2.55^Ac^ ± 0.51	2.57^Ac^ ± 0.81

*Average values followed by different capital letters in the same column or by different lowercase letters in the same line differ according to Tukey's test (P < 0.05)*.

The DP caused a significant decrease in serum potassium, chloride and ionized calcium in comparison to baseline values (T-36h). Serum potassium returned to baseline during the fluid therapy phase in all three treatment groups at T6h and T8h (Table 2). Serum chloride returned to baseline in the EsHYPO and RL IV group and values above baseline were observed in the EsISO group at T4h, T6h, and T8h. Ionized calcium returned to baseline in the EsHYPO and EsISO treatment groups but remained lower than baseline in the LR IV treatment group (Table 2).

A significant increase in serum magnesium, phosphorus, urea, creatinine, lactate, glucose, and osmolality in comparison to baseline values (T-36h) was noted after the DP (Tables 2, 3). During the fluid therapy phase magnesium decreased even further in all three groups and more profoundly in the LR IV treatment at T8h. Phosphorus returned to baseline in the EsISO and EsHYPO treatment groups but remained higher than baseline in the RL IV group. Glucose levels were significantly higher than baseline during the first hours of EsISO and EsHYPO treatments, returning to baseline at T8h on both groups. Glucose levels remained in the reference range during the entire fluid therapy phase in the LR IV treatment group (Table 3). All three treatments were effective in correcting urea, creatinine, lactate and osmolarity values back to baseline (Tables 2, 3).

**Table 3:** Means and standard deviations of serum urea (mg dl^−1^), plasma lactate (mmol L^−1^), serum creatinine (mg dl^−1^), plasma glocose (mg dl^−1^), and serum osmolarity (mOsm L^−1^) in horses submitted to water and food restriction and treated in a 6 × 3 crossover design with different hydroelectrolytic therapy modalities.

**Treatment**	**Time**					
	**(-)36 h**	**0 h**	**2 h**	**4 h**	**6 h**	**8 h**
**Serum urea**						
EsHYPO	25.83^Ab^ ± 9.77	51.33^Aa^ ± 9.22	51.17^Aa^ ± 10.25	47.67^Aa^ ± 8.09	43.67^Aa^ ± 7.74	38.50^Ab^ ± 5.96
EsISO	25.83^Ab^ ± 3.31	49.33^Aa^ ± 5.57	50.33^Aa^ ± 7.45	45.33^Aa^ ± 7.26	38.67^Aa^ ± 6.92	35.33^Ab^ ± 4.68
LR IV	25.67^Ab^ ± 9.05	52.00^Aa^ ± 14.09	50.67^Aa^ ± 9.11	50.50^Aa^ ± 11.61	44.33^Aa^ ± 7.28	38.00^Ab^ ± 11.10
**Plasma lactate**						
EsHYPO	2.18^Ab^ ± 0.88	4.13^Aa^ ± 1.89	3.23^Aa^ ± 0.55	3.53^Aa^ ± 1.12	2.47^Bb^ ± 0.56	2.55^Ab^ ± 0.65
EsISO	2.32^Ab^ ± 0.59	4.18^Aa^ ± 1.40	3.08^Aa^ ± 1.08	2.78^Ab^ ± 0.55	2.55^Bb^ ± 0.29	2.67^Ab^ ± 0.50
LR IV	1.85^Ab^ ± 0.41	4.73^Aa^ ± 0.58	3.95^Aa^ ± 0.53	3.58^Aa^ ± 0.62	3.52^Ab^ ± 0.53	2.58^Ab^ ± 0.62
**Serum creatinine**						
EsHYPO	1.27^Ac^ ± 0.12	1.92^Aa^ ± 0.15	1.65^Ab^ ± 0.16	1.67^Ab^ ± 0.22	1.55^Ab^ ± 0.08	1.28^Ac^ ± 0.17
EsISO	1.28^Ac^ ± 0.18	1.95^Aa^ ± 0.18	1.65^Ab^ ± 0.32	1.63^Ab^ ± 0.27	1.43^Ab^ ± 0.34	1.25^Ac^ ± 0.31
LR IV	1.10^Ac^ ± 0.13	1.82^Aa^ ± 0.12	1.43^Bb^ ± 0.15	1.32^Bb^ ± 0.16	1.32^Ab^ ± 0.19	1.22^Ac^ ± 0.17
**Plasma glucose**						
EsHYPO	96.00^Ac^ ± 5.10	111.17^Ab^ ± 15.00	149.00^Ba^ ± 20.99	142.33^Ba^ ± 11.36	120.33^Ba^ ± 10.58	102.17^Ac^ ± 18.71
EsISO	92.50^Ac^ ± 5.68	104.67^Ab^ ± 11.15	178.50^Aa^ ± 22.12	184.83^Aa^ ± 29.94	148.17^Aa^ ± 28.53	117.17^Ac^ ± 21.49
LR IV	90.83^Ac^ ± 5.04	101.83^Aa^ ± 7.83	97.83^Ca^ ± 5.04	97.67^Ca^ ± 3.14	99.33^Bb^ ± 3.93	104.50^Aa^ ± 7.34
**Serum osmolarity**						
EsHYPO	266.50^Ab^ ± 16.84	280.00^Aa^ ± 13.49	xxx	271.00^Ab^ ± 11.59	xxx	269.50^Ab^ ± 10.60
EsISO	269.83^Ab^ ± 7.91	279.00^Aa^ ± 3.41	xxx	271.00^Ab^ ± 6.87	xxx	271.83^Ab^ ± 5.12
LR IV	270.17^Ab^ ± 12.51	281.33^Aa^ ± 9.91	xxx	277.00^Ab^ ± 6.96	xxx	273.50^Ab^ ± 7.12

*Average values followed by different capital letters in the same column or by different lowercase letters in the same line differ according to Tukey's test (P < .05)*.

A significant increase in urinary sodium, potassium, magnesium, creatinine and urea in comparison to baseline values (T-36h) was noted after the DP (Tables 4, 5). Urinary sodium decreased in the EsHYPO and EsISO groups and increased in the LR IV group during the fluid therapy phase (T4h, T6h, and T8h). The DP caused a significant decrease in urinary chloride and calcium. Urinary chloride remained low throughout the fluid therapy phase in the EsISO and EsHYPO treatment groups, while a gradual increase was noted in the LR IV treatment starting at T6h (Table 4). Urinary potassium also decreased in all three groups during the fluid therapy phase (Table 4). Urinary calcium remained lower than baseline during the fluid therapy phase in all three treatment groups (Table 4). Urinary magnesium returned to baseline values at T8h in the EsISO and EsHYPO treatment groups and values below baseline were noted in the LR IV treatment group at T8h (Table 4). Urinary urea and creatinine decreased over the hydration phase in all three treatment groups (Table 5).

**Table 4:** Means and standard deviations of urinary sodium (mmol L^−1^), potassium (mmol L^−1^), chloride (mmol L^−1^), urinary calcium (mg dl^−1^) and magnesium (mg dl^−1^) in horses submitted to water and food restriction and treated in a 6 × 3 crossover design with different hydroelectrolytic therapy modalities.

**Treatment**	**Time**					
	**(-)36 h**	**0 h**	**2 h**	**4 h**	**6 h**	**8 h**
**Urinary sodium**						
EsHYPO	13.33^Ab^ ± 11.78	42.33^Aa^ ± 38.09	22.50^Aa^ ± 29.15	8.00^Bb^ ± 11.63	6.17^Bb^ ± 8.28	14.17^Bb^ ± 11.55
EsISO	14.33^Ab^ ± 19.33	47.50^Aa^ ± 41.13	20.33^Aa^ ± 34.45	16.33^Bb^ ± 26.70	21.17^Bb^ ± 26.12	21.17^Bb^ ± 28.92
LR IV	8.17^Ac^ ± 6.24	61.00^Ab^ ± 39.51	59.33^Ab^ ± 69.40	106.67^Aa^ ±70.67	153.67^Aa^ ± 28.39	140.83^Aa^ ± 36.5
**Urinary potassium**						
EsHYPO	90.83^Ab^ ± 97.66	206.67^Aa^ ± 173.97	157.17^Aa^ ± 138.21	49.50^Bb^ ± 61.41	22.83^Ab^ ± 26.03	14.00^Ac^ ± 4.82
EsISO	96.17^Ab^ ± 100.62	171.00^Aa^ ± 95.79	114.00^Aa^ ± 66.14	74.17^Bb^ ± 53.17	34.00^Ab^ ± 25.64	12.50^Ac^ ± 8.31
LR IV	84.00^Ab^ ± 89.02	194.00^Aa^ ± 162.36	128.67^Aa^ ± 110.64	120.33^Aa^ ±94.80	34.67^Aa^ ± 27.08	10.00^Ac^ ± 4.34
**Urinary chloride**						
EsHYPO	96.67^Aa^ ± 45.34	31.67^Ab^ ± 20.78	27.00^Ab^ ± 14.89	25.50^Ab^ ± 17.63	25.17^Bb^ ± 23.57	26.17^Bb^ ± 8.47
EsISO	97.17^Aa^ ± 24.29	29.17^Ab^ ± 26.66	38.67^Ab^ ± 16.52	50.33^Ab^ ± 32.28	51.50^Ab^ ± 29.32	37.00^Bb^ ± 24.50
LR IV	81.67^Ab^ ± 70.01	27.00^Ac^ ± 24.86	31.00^Ac^ ± 23.14	36.17^Ac^ ± 28.10	71.67^Ab^ ± 29.40	102.67^Aa^ ± 44.47
**Urinary calcium**						
EsHYPO	40.15^Aa^ ± 9.28	20.20^Ab^ ± 16.09	11.15^Ab^ ± 4.08	15.90^Ab^ ± 14.55	23.57^Ab^ ± 12.75	7.77^Ac^ ± 3.89
EsISO	37.58^Aa^ ± 6.36	21.30^Ab^ ± 12.28	24.20^Ab^ ± 15.02	19.98^Ab^ ± 16.28	14.68^Ab^ ± 3.78	7.72^Ac^ ± 5.95
LR IV	35.28^Aa^ ± 13.46	21.47^Ab^ ± 5.91	13.45^Ab^ ± 3.98	5.67^Bc^ ± 4.26	6.13^Bc^ ± 2.67	4.87^Bc^ ± 1.13
**Urinary magnesium**						
EsHYPO	2.96^Ab^ ± 1.44	4.67^Aa^ ± 1.97	6.29^Aa^ ± 2.08	5.99^Aa^ ± 2.04	6.58^Aa^ ± 1.75	4.81^Ab^ ± 2.95
EsISO	4.20^Ab^ ± 1.44	5.33^Aa^ ± 0.92	4.88^Aa^ ± 1.50	6.07^Aa^ ± 0.88	5.22^Aa^ ± 1.07	3.52^Ab^ ± 0.87
LR IV	2.87^Ab^ ± 1.74	5.08^Aa^ ± 0.77	3.12^Bb^ ± 1.11	3.90^Ab^ ± 1.07	3.32^Bb^ ± 1.46	1.62^Bc^ ± 0.66

*Average values followed by different capital letters in the same column or by different lowercase letters in the same line differ according to Tukey's test (P < 0.05)*.

**Table 5:** Means and standard deviations of urinary urea (mg dl^−1^), creatinine (mg dl^−1^) and glucose (mg dl^−1^) in horses submitted to water and food restriction and treated in a 6 × 3 crossover design with different hydroelectrolytic therapy modalities.

**Treatment**	**Time**					
	**(-)36 h**	**0 h**	**2 h**	**4 h**	**6 h**	**8 h**
**Urinary urea**						
EsHYPO	156.83^Ac^ ± 46.24	410.50^Ba^ ± 16.90	370.50^Ba^ ± 47.93	269.50^Bb^ ± 73.24	218.33^Bb^ ± 61.70	146.00^Ac^ ± 59.90
EsISO	172.83^Ab^ ± 73.89	477.83^Ba^ ± 71.72	374.00^Ba^ ±150.23	354.67^Ba^ ±107.49	314.00^Ba^ ± 81.20	172.17^Ab^ ± 38.84
LR IV	224.33^Ab^ ±104.24	627.67^Aa^ ± 55.01	628.17^Aa^ ± 63.36	606.17^Aa^ ± 100.78	467.50^Aa^ ±170.17	166.67^Ab^ ± 92.07
**Urinary creatinine**						
EsHYPO	39.58^Ab^ ± 29.93	472.50^Aa^ ±119.25	437.50^Aa^ ±241.52	236.67^Aa^ ±123.59	33.07^Bb^ ± 26.26	10.83^Ac^ ± 8.61
EsISO	39.17^Ab^ ± 35.97	340.42^Aa^ ±123.99	221.25^Aa^ ± 79.13	225.00^Aa^ ± 90.80	85.00^Ab^ ± 53.22	21.67^Ab^ ± 20.17
LR IV	39.58^Ab^ ± 54.28	347.08^Aa^ ±112.79	282.92^Aa^ ±124.98	212.92^Aa^ ±147.72	37.05^Bb^ ±18.24	8.75^Ac^ ± 6.85
**Urinary glucose**						
EsHYPO	0.00^Ab^ ± 0.00	0.00^Ab^ ± 0.00	0.00^Bb^ ± 0.00	0.33^Ba^ ± 0.82	0.00^Bb^ ± 0.00	0.17^Ba^ ± 0.41
EsISO	0.00^Ac^ ± 0.00	0.00^Ac^ ± 0.00	0.17^Ab^ ± 0.41	1.00^Aa^ ± 1.26	1.17^Aa^ ± 1.33	0.33^Ab^ ± 0.52
LR IV	0.00^Aa^ ± 0.00	0.00^Aa^ ± 0.00	0.00^Ba^ ± 0.00	0.00^Ca^ ± 0.00	0.00^Ca^ ± 0.00	0.00^Ca^ ± 0.00

*Average values followed by different capital letters in the same column or by different lowercase letters in the same line differ according to Tukey's test (P < 0.05)*.

Groups treated with enteral electrolyte solutions, which had the addition of energy sources, demonstrated an increase in urinary glucose after T2h, with EsISO treatment animals showing the highest values (Table 5).

During the fluid therapy phase all treatments caused a gradually increase in urine volume from T4h to T8h, with the highest volume observed in the LR IV treatment group at T8h. The DP did not affect urinary pH. Significantly higher pH values in comparison to baseline (T-36h) were observed only in the LR IV group at T4h and T6h. The urine specific gravity suffered a significant increase in comparison to baseline (T-36h). All treatments returned urine specific gravity values to baseline values (Table 6).

**Table 6:** Means and standard deviations of urinary volume (ml), pH and specific gravity in horses submitted to water and food restriction and treated in a 6 × 3 crossover design with different hydroelectrolytic therapy modalities.

**Treatment**	**Time**					
	**(-)36 h**	**0 h**	**2 h**	**4 h**	**6 h**	**8 h**
**Urinary volume**						
EsHYPO			110.00^Ad^ ± 67.90	1376.67^Ac^ ± 1163.9	3095.8^Ab^ ± 2296.51	6611.6^Ba^ ± 2384.28
EsISO			97.50^Ad^ ± 73.06	675.00^Ac^ ± 413.22	1950.0^Ab^ ±1017.84	5785.8^Ba^ ± 2036.25
LR IV			135.00^Ad^ ± 63.48	836.67^Ac^ ± 900.81	2100.0^Ab^ ± 1764.55	10775^Aa^ ± 4534.29
**Urinary pH**						
EsHYPO	7.25^Aa^ ± 1.08	7.50^Aa^ ± 1.18	7.83^Aa^ ± 1.13	7.58^Ba^ ± 1.02	7.50^Ba^ ± 0.89	7.00^Aa^ ± 1.22
EsISO	7.25^Aa^ ± 0.88	7.58^Aa^ ± 0.97	7.83^Aa^ ± 0.98	7.75^Ba^ ± 0.94	7.42 ^Ba^ ± 0.97	7.08^Aa^ ± 0.92
LR IV	7.42^Ab^ ± 1.11	7.50^Ab^ ± 1.18	7.50^Ab^ ± 1.18	8.08^Aa^ ± 1.07	8.33^Aa^ ± 0.41	7.67^Ab^ ± 1.03
**Specific gravity**						
EsHYPO	1010.0^Ab^ ± 6.81	1031.6^Aa^ ± 7.74	1030.0^Aa^ ± 12.52	1019.3^Aa^ ± 14.57	1007.0^Ab^ ± 4.86	1004.1^Ab^ ± 2.86
EsISO	1007.6^Ac^ ± 6.12	1025.0^Aa^ ± 10.64	1025.6^Aa^ ± 8.62	1026.3^Aa^ ± 5.13	1013.0^Ab^ ± 7.35	1004.0^Ac^ ± 2.53
LR IV	1007.6^Ac^ ± 4.46	1031.0^Aa^ ± 11.71	1025.6^Aa^ ± 8.71	1024.6^Aa^ ± 11.15	1016.0^Ab^ ± 12.46	1005.6^Ac^ ± 2.94

*Average values followed by different capital letters in the same column or by different lowercase letters in the same line differ according to Tukey's test (P < 0.05)*.

A significant effect of the DP on acid base balance was noted and characterized by an increase in pH, $\text{HC}{\text{O}}_{3}^{-}$ and BE. Likewise, SID values increased after DP. The three treatments caused a decrease in pH, $\text{HC}{\text{O}}_{3}^{-}$, BE and SID, while pCO_2_ and AG values throughout the experimental phase showed little variation in all treatment groups (Table 7).

**Table 7:** Means and standard deviations of blood pH, $\text{cHC}{\text{O}}_{3}^{-}$ (mmol L^−1^), pCO_2_ (mmHg), Base Excess (BE) (mmol L^−1^), Strong Ions Difference (SID) (mEq L^−1^) and Anion Gap (AG) (mEq L^−1^) of horses submitted to water and food restriction and treated in a 6 × 3 crossover design with different hydroelectrolytic therapy modalities.

**Treatment**	**Time**					
	**(-)36 h**	**0 h**	**2 h**	**4 h**	**6 h**	**8 h**
**Blood pH**						
EsHYPO	7.41^Ab^ ± 0.02	7.43^Aa^ ± 0.02	7.39^Ac^ ± 0.02	7.37^Ac^ ± 0.02	7.37^Ac^ ± 0.02	7.36^Bc^ ± 0.03
EsISO	7.41^Ab^ ± 0.02	7.45^Aa^ ± 0.02	7.39^Ac^ ± 0.03	7.36^Ad^ ± 0.03	7.35^Ad^ ± 0.04	7.33^Bd^ ± 0.04
LR IV	7.40^Ab^ ± 0.02	7.43^Aa^ ± 0.02	7.41^Ab^ ± 0.03	7.39^Ab^ ± 0.03	7.39^Ab^ ± 0.02	7.39^Ab^ ± 0.02
**$\text{cHC}{\text{O}}_{3}^{-}$**						
EsHYPO	27.48^Ab^ ± 2.37	30.82^Aa^ ± 2.45	27.88^Ab^ ± 3.10	26.25^Ab^ ± 2.66	24.85^Ac^ ± 2.31	23.85^Bc^ ± 2.07
EsISO	28.67^Ab^ ± 1.89	32.58^Aa^ ± 1.77	28.93^Aa^ ± 2.40	26.02^Ac^ ± 2.19	24.28^Ac^ ± 2.56	23.43^Bc^ ± 2.91
LR IV	27.82^Ab^ ± 1.83	29.45^Aa^ ± 2.38	29.13^Ab^ ± 2.91	26.48^Ab^ ± 1.20	26.05^Ab^ ± 0.83	27.40^Ab^ ± 1.85
**PCO**_**2**_						
EsHYPO	43.30^Aa^ ± 2.73	45.70^Aa^ ± 2.07	46.02^Aa^ ± 4.23	44.47^Aa^ ± 3.70	42.78^Aa^ ± 3.71	41.90^Aa^ ± 3.19
EsISO	44.47^Ab^ ± 1.89	47.05^Aa^ ± 1.97	46.75^Ab^ ± 1.84	45.38^Ab^ ± 2.10	43.43^Ab^ ± 1.99	44.07^Ab^ ± 2.32
LR IV	44.48^Aa^ ± 1.87	44.33^Aa^ ± 1.94	45.32^Aa^ ± 4.42	43.45^Aa^ ± 1.55	42.78^Aa^ ± 2.44	44.82^Aa^ ± 2.55
**BE**						
EsHYPO	2.67^Aa^ ± 2.73	6.67^Ba^ ± 2.73	3.17^Aa^ ± 3.31	1.17^Ab^ ± 2.99	−0.33^Ab^ ± 2.50	−1.50^Aa^ ± 2.43
EsISO	4.00^Ab^ ± 2.19	9.17^Aa^ ± 1.83	4.17^Ab^ ± 2.86	0.67^Ac^ ± 2.50	−1.17^Ac^ ± 3.31	−2.33^Ab^ ± 3.44
LR IV	2.67^Aa^ ± 1.86	6.00^Ba^ ± 1.55	4.50^Aa^ ± 3.21	1.67^Ac^ ± 1.63	1.17^Ac^ ± 0.98	2.33^Aa^ ± 1.75
**SID**						
EsHYPO	39.80^Ab^ ± 1.72	41.72^Aa^ ± 2.48	36.88^Bc^ ± 2.73	36.58^Bc^ ± 1.43	31.18^Bd^ ± 2.71	36.77^Ac^ ± 0.72
EsISO	37.30^Ab^ ± 2.99	41.33^Aa^ ± 5.93	35.87^Bb^ ± 1.85	33.90^Cc^ ± 2.47	34.23^Bc^ ± 2.07	32.85^Bc^ ± 1.99
LR IV	37.57^Ab^ ± 4.22	43.30^Aa^ ± 3.46	40.67^Aa^ ± 2.42	39.98^Aa^ ± 4.14	40.55^Aa^ ± 3.60	37.48^Aa^ ± 3.38
**AG**						
EsHYPO	12.32^Aa^ ± 2.42	10.90^Ab^ ± 2.94	9.00^Ab^ ± 1.47	10.33^Ab^ ± 2.63	6.33^Ac^ ± 2.64	12.92^Aa^ ± 2.32
EsISO	8.63^Aa^ ± 1.81	8.75^Aa^ ± 4.66	6.93^Bc^ ± 2.17	7.88^Bc^ ± 1.61	9.95^Bb^ ± 2.73	9.42^Ab^ ± 1.69
LR IV	9.75^Ac^ ± 3.96	13.85^Ab^ ± 3.41	11.53^Cb^ ± 2.60	13.50^Cb^ ± 3.74	14.50^Ca^ ± 3.58	10.08^Ac^ ± 4.05

*Average values followed by different capital letters in the same column or by different lowercase letters in the same line differ according to Tukey's test (P < 0.05)*.

## Discussion

Serum sodium was not affected by the experimental dehydration protocol and during the fluid therapy phase in the EsHYPO and EsISO groups (Table 2). Therefore, the hypotonic enteral solution used in the present study is safe to rehydrate horses without causing hyponatremia, which is the major concern with this type of fluid. The DP caused an increase in urinary sodium (T0h) in comparison to baseline (T-36h), but during the fluid therapy phase values returned to baseline in the EsHYPO and EsISO treatments at T4h (Table 4). This was possibly a result of the composition of the electrolyte solutions associated with an increase in urinary output due to fluid therapy and urine dilution and decreased urinary sodium excretion. Similar results have been reported in adult horses (5). However, weaned foals treated with enteral electrolyte solutions for 12 h at the same infusion rate used in the present study showed an increase in urinary sodium excretion (4).

Serum sodium levels did not significantly change within the LR IV group during the fluid therapy phase but were significantly higher than those observed in the EsISO and EsHYPO treatments at T2h, T4h, and T6h (Table 2). This can be attributed to the composition of lactate Ringer's solution (LRS), which contains more sodium than the enteral electrolyte solutions used in the present assay. This effect was transient and lacks clinical relevance, since sodium concentrations in the LR IV group did not exceed the normal range for horses (8). In addition, urinary sodium increased significantly (*P* < 0.05) during the fluid therapy phase in RL IV groups, indicating that the amount of sodium in the lactate Ringer's solution, despite being physiological, is high (Table 4). Similar results were observed in neonatal foals (9).

Serum potassium decreased following the DP (*P* < 0.05). Since forages are important sources of potassium (10), food restriction might have contributed to this finding. Also, furosemide increases renal excretion of potassium (11). During the fluid therapy phase from T6h on, all treatments were effective in bringing potassium levels back to baseline (Table 2), likely due to the presence of this electrolyte in all three fluids. An increase in urinary potassium was also observed after the DP, mainly due to the action of furosemide (Table 4). A gradual decrease was observed during the fluid therapy phase and values significantly lower than baseline were noted at T8h. As mentioned by Harrison-Bernard (12), the decrease in urinary potassium excretion may be associated with the plasma volume expansion caused by fluid therapy, reducing renal potassium excretion.

Serum chloride values decreased significantly during the DP (T0h), which is expected due to the action of furosemide (11). This phenomenon was not observed in another experiment where the dehydration induction protocol consisted of only 24 h of water and food deprivation combined with only one dose of furosemide 3 mg kg^−1^ of furosemide (13). All three fluids were able to reverse serum chloride depletion during the fluid therapy phase (Table 2), with the EsISO enteral treatment showing values above baseline at the end of the trial but still within the normal range (8). Despite containing more chloride than the enteral solutions, LR IV treatment did not promote an increase in serum values of this electrolyte. This can be justified by the fact that LR contains a similar amount of chloride as equine serum or plasma (105 mmol L^−1^). Also, the RL IV group showed greater urinary chloride excretion, sustaining its serum levels unchanged. In order to maintain serum chloride levels, a lower urinary chloride excretion was noted in the EsHYPO and EsISO treatment groups (Table 4).

Serum ionized calcium decreased after the DP, mainly because of fasting and the diuretic action of furosemide, since horses' urine is rich in calcium (14). Both enteral therapies (EsISO and EsHYPO) were more efficient in maintaining serum levels of this element (Table 2). Serum ionized calcium remained below baseline at the end of the experimental period (T8h). Urinary Ca^++^ excretion also decreased in the DP and remained low during the hydration phase in the RL IV group (Table 4). This can be justified by the fact that lactated Ringer's solution (LRS) contains lower calcium levels than equine plasma (1). Also, it has been described that LRS solution does not have an adequate concentration of calcium (15). Since calcium is naturally excreted in horses' urine, its supplementation is necessary when using LRS intravenously, especially in long-term fluid therapy and in patients who are not eating. The EsHYPO and EsISO enteral solutions had significant amounts of calcium acetate (1g.L^−1^). This justifies the effectiveness of these solutions in reversing the decrease in serum ionized calcium caused by the DP. Urinary calcium excretion decreased at T0h (*P* < 0.05) and remained low throughout the fluid therapy phase in the EsHYPO and EsISO groups. Although lower than baseline, values were significantly higher than those of the LR IV treatment, demonstrating the effect of higher calcium concentration in the enteral electrolyte solutions.

Serum magnesium increased after the DP (*P* < 0.05) likely due to renal compensatory mechanisms that acted to preserve it in exchange for the excretion of cations stimulated by furosemide. All three treatments reversed this increase by replacing blood volume, however, in the LR IV group there was a marked and gradual decrease in this electrolyte, reaching its lowest value at T8h (Table 2). This is justified by the absence of magnesium sources in LRS and by hemodilution. The absence of magnesium in LRS also explains its lower urinary excretion in the LR IV treatment group in comparison to both enteral fluids (Table 4). A slight decrease in serum magnesium was observed in both enteral electrolyte treatment groups despite the addition of this electrolyte in their composition. This was possibly due to hemodilution and the amount of magnesium chloride contained in both solutions, as mentioned by Ribeiro Filho et al. (5). Although low, this change was significantly milder when compared with the LR IV group. Urinary magnesium at some time points during the fluid therapy phase with EsHypo and EsISO reached values above baseline (T-36h). This constitutes an advantage in the administration of enteral electrolyte solutions containing magnesium, especially in patients with hypomagnesaemia.

Serum phosphorus showed different behavior for each experimental group, since neither enteral solutions nor LRS contain this element. Therefore, the dynamics of its values are due to the hemodilution caused by fluid replacement as observed by Ribeiro Filho et al. (16, 17), associated with natural endocrine mechanisms of organic phosphorus regulation (1).

In the present study, both urea and creatinine increased after the DP demonstrating decreased blood volume and glomerular filtration rate, even though values did not exceed the reference intervals for the species (8). Thus, the DP was able to promote dehydration in the mares without causing pre-renal azotemia (14). Urea and creatinine values progressively regressed to baseline after 8 h of treatment in all experimental groups, demonstrating similar efficacy in restoring blood volume, tissue perfusion and glomerular filtration. However, LR IV treatment was more effective in decreasing creatinine values faster than both enteral electrolyte treatments (T2h and T4h) (Table 3). This result shows that the administration of electrolyte solutions intravenously expands blood volume quickly, promoting the excretion of undesirable metabolites. However, from T6h on, both enteral electrolyte solutions had the same effect as the RL IV group. The dynamics of urinary urea and creatinine levels reflected a marked urine concentration in the DP. All treatments, although in different ways, were able to dilute urine throughout the hydration period (Table 5). The dynamics of urinary urea and creatinine values proved the efficacy of enteral fluid therapy in continuous flow (EFTcf) compared to LR IV treatment in correcting urine concentration. Similar results have been observed after 12 h of EFTcf at similar infusion rates in horses (5).

In the present study, plasma lactate values significantly increased after the DP (*P* < 0.05), confirming decreased blood volume and tissue perfusion. All three treatments were similarly effective in bringing lactate levels back to baseline, demonstrating equivalent efficacy in promoting volume expansion and tissue perfusion (Table 3). The effect of different formulations of EFTcf in reducing serum lactate in horses by expanding plasma volume and promoting tissue perfusion has been previously reported (17).

The DP promoted a significant increase in plasma glucose in all three treatment groups (*P* < 0.05). This can be attributed to fasting, which induces gluconeogenesis by recruiting body energy reserves (18) and by the stress promoted by the DP (19). Glucose behaved differently in all three treatment groups during the fluid therapy phase, which can be explained by the formulation of each solution. LRS has no energy sources, thus the LR IV group demonstrated significantly lower blood glucose during the hydration phase when compared to the EsISO and EsHYPO groups, but still within the normal range. EsISO and EsHYPO groups had significantly higher values of plasma glucose, specially EsISO when compared to EsHYPO (Table 3). This difference can be justified by the greater amount of energy sources in the EsISO formulation. Results demonstrated the action of glycemic regulatory mechanisms in all groups. The efficacy in restoring glycemia of enteral solutions associated with highly metabolizable energy sources has been previously observed in studies that compared them to other types of fluid therapies such as LRS (6) and that evaluated different formulations of EFTcf (17). Thus, in the current experimental design, the addition of energy sources in enteral solutions formulations for hydroelectrolytic therapy in horses is advantageous over LR IV therapy.

The dynamics of serum osmolarity values illustrate plasma expansion and blood volume restauration in the different fluid therapy modalities studied. All three treatments were efficient in reversing the increase in serum osmolarity that occurred due to food and water deprivation and water loss induced by the DP, leading to higher concentrations of serum solutes. There was a decrease in serum osmolarity values during the hydration phase, but at T8h values remained similar to baseline (Table 3). This was considered an advantage, since the main concern with the use of hypotonic enteral electrolyte solutions in horses is the decrease in serum osmolarity. The application of hypotonic enteral electrolyte solutions has already been tested in several animal species without triggering this adverse effect (4, 5, 20).

All treatments promoted an increase in urinary volume starting at T2h, accompanied by changes in urinary color from dark amber at T2h and T4h to a clear, translucent and limpid urine at T6h and T8h. The LR IV treatment promoted significantly greater urination volume when compared to the EsISO and EsHYPO treatments. This difference is justified by the fact that the LR IV treatment is administered directly into the vascular bed, which increases its speed and capacity to promote blood volume expansion and glomerular filtration rate. In addition, it stimulates the release of the atrial natriuretic peptide and all these mechanisms translate into large volumes of urine (1). This can be seen as an advantage of LR IV therapy, especially in the replacement phase of a therapeutic fluid therapy plan. Nevertheless, this significantly higher urine volume demonstrates that the IV fluids are excreted shortly after administration, not allowing time for the transfer of fluids from the vascular compartment to the tissues and the intracellular compartment, which is the main objective when hydrating an equine patient (21). Therapies with enteral electrolyte solutions also induced urination, but in significantly smaller volumes. Since the administered volumes of electrolyte solutions were equivalent between EsISO, EsHYPO and LR IV groups, the smaller amount of urine excreted demonstrates that EFTcf, in addition to blood volume expansion and increase in glomerular filtration rate, allows greater retention of the administered fluids. This may yield better distribution of water and electrolytes into tissues, intracellular compartments and also the rehydration of ingesta. EFTcf's ability to promote diuresis was also reported by Monteiro et al. (4) and Ribeiro Filho et al. (5).

No changes in urinary pH were observed after the DP and within the EsISO and EsHYPO treatment groups during the fluid therapy phase. Urinary pH was significantly higher in the RL IV group in comparison to both EFTcf at T4h and T6h (Table 6), but values remained in the normal range (22). Mild aciduria was previously described in horses treated with different EFTcf formulations due to renal mechanisms to control the acid-base balance, mainly by the renal excretion of H^+^ ions (3).

Urinary specific gravity, a sensitive marker of blood volume, increased as a consequence of the DP (*P* < 0.05), but all three treatments were similarly effective to promote the production of diluted urine, leading to hypostenuria at the end of the 8-h hydration period. The dynamics of urinary specific gravity values observed here confirms the effectiveness of EFTcf therapies compared to LR IV treatment in restoring hydration. Other studies showed similar findings in horses treated with different formulations of enteral fluid therapies (3–5).

Groups treated with enteral electrolyte solutions, which have the addition of energy sources, presented mild glycosuria at a few time points. This finding was more remarkable in EsISO, which had a higher glycide content. According to Wilson (14), the presence of glucose in urine reflects an overload on renal function's ability to reabsorb filtered blood glucose and can occur with the use of fluid therapies with carbohydrate excess (5). Since the glycosuria observed in the present study was discrete (14) it was not considered clinically relevant (Table 5).

The DP caused an increase in pH, HCO${}_{3}^{-}$ and BE (Table 7). This was determined by the chloride depressant action of furosemide, as reported by Freestone et al. (23) and Alves et al. (15), who also used this drug in the experimental induction of hydroelectrolytic and acid-base imbalances in horses. The excretion of chloride is compensated by the retention of bicarbonate, which justifies the changes observed. Depending on the posology furosemide may cause hypochloremic metabolic alkalosis. All treatments were able to reverse the changes mentioned above, however the EsISO and EsHYPO treatment groups reached the lowest pH, HCO${}_{3}^{-}$ and BE levels at T8h when compared to T-36h. This effect was not observed in the LR IV group (Table 7).

The energy source of EsHYPO and EsISO treatments may have been the cause of the slight decrease in blood pH, HCO${}_{3}^{-}$ and BE observed. As mentioned by Gomes et al. (24), the addition of carbohydrates in enteral solutions can lead to intestinal microbial fermentation and the production of organic acids, that are absorbed and might generate this effect. Even so, it is necessary to highlight that the effect of enteral electrolyte solutions on the referred variables was mild, especially in the EsHYPO group. In addition, none of the animals treated with enteral electrolyte solutions showed any signs or complications related to these findings. The small acidifying effect observed in the EsISO group makes this solution an option for dehydrated horses with mild metabolic alkalosis.

No significant changes in pH, HCO${}_{3}^{-}$ and BE were detected during the hydration period in LR IV treatment group. Since Lactate Ringer's solution has a similar composition to plasma it does not have an alkalizing or acidifying effect. Corroborating this fact, it was observed that the intravenous use of LRS in a volume of 10% of body weight for a 6-h treatment in healthy horses did not induce changes in acid-base balance (25). Likewise, daily volumes of 12 to 19% of body weight of intravenous lactate Ringer's solution infused in horses submitted to experimental induction of colonic impaction had no significant effect on the acid base balance (26).

PCO_2_ values were significantly increased in the EsISO group at T0h (*P* < 0.05), which characterizes the development of respiratory compensatory activity. Although minor, according to Hinchcliff et al. (27) it already expresses mild respiratory acidosis. At T0h BE demonstrated low-intensity metabolic alkalosis (9.17 mmol L^−1^), so the respiratory system compensates with respiratory acidosis (28). During the fluid therapy period, secondary or compensatory respiratory acidosis resolved with pCO_2_ values returning to baseline at T8h (Table 7). In the EsHYPO and LR IV treatments, pCO_2_ remained unchanged throughout experimental phase (*P* > 0.05) confirming that both did not cause changes that required compensation in the acid-base balance.

The anion gap showed little variation throughout the experimental period and values remained in the normal range (24, 26) in all three treatment groups. SID remained unchanged in the LR IV treatment group during the fluid therapy period and at the lower limit in EsHYPO. EsISO contains more chloride than the other solutions, therefore it promoted the greatest decrease in SID values at T8h, revealing metabolic acidosis.

Enteral solutions can be used in horses as maintenance fluid therapy, especially given its physiological route of administration and its low cost, it also can be manipulated according to the patient needs. The use of LR IV is still necessary in cases where the horse has reflux, intestinal obstruction and when it is not able to stand. EsHYPO is a good maintenance choice for long term fluid therapy, while EsISO would be better used as treatment for mild alkalosis.

## Conclusion

The present study demonstrated the efficacy and safety of enteral fluid therapy with isotonic and hypotonic electrolyte solution administered in continuous flow in the replacement of blood volume in horses when compared to intravenous fluid therapy with lactated Ringer's solution (RL IV). Hypotonic enteral solution (EsHYPO) and lactated Ringer's solution (RL IV) can be used in long-term fluid therapy. Since it has a slightly acidifying effect, the isotonic enteral solution (EsISO) could be an option in the treatment of low-intensity metabolic alkalosis.

## Data Availability Statement

The raw data supporting the conclusions of this article will be made available by the authors, without undue reservation.

## Ethics Statement

The animal study was reviewed and approved by Animal Use Ethics Committee of Universidade Federal de Viçosa (UFV).

## Author Contributions

DD, JR, TB, and RV were responsible for the conception of the study, data analysis, and provided intellectual input on the manuscript. DD, JR, NM, FD, SA, and PS were responsible for carrying out the experimental phase of the study. DD, JR, RT, HM, and NM were responsible for data interpretation and writing of the manuscript. All authors contributed to the article and approved the submitted version.

## Conflict of Interest

FD is employed by the company Crescer Consultancy Service. The remaining authors declare that the research was conducted in the absence of any commercial or financial relationships that could be construed as a potential conflict of interest.

## Publisher's Note

All claims expressed in this article are solely those of the authors and do not necessarily represent those of their affiliated organizations, or those of the publisher, the editors and the reviewers. Any product that may be evaluated in this article, or claim that may be made by its manufacturer, is not guaranteed or endorsed by the publisher.
